# Targeting deubiquitinating enzymes in cancer stem cells

**DOI:** 10.1186/s12935-017-0472-0

**Published:** 2017-11-03

**Authors:** Hu Lei, Huizhuang Shan, Yingli Wu

**Affiliations:** Hongqiao International Institute of Medicine, Shanghai Tongren Hospital/Faculty of Basic Medicine, Chemical Biology Division of Shanghai Universities E-Institutes, Key Laboratory of Cell Differentiation and Apoptosis of the Chinese Ministry of Education, Shanghai Jiao Tong University School of Medicine, Shanghai, 200025 China

**Keywords:** Cancer stem cells, Deubiquitinating enzymes, Cancer therapies, CSCs

## Abstract

Cancer stem cells (CSCs) are rare but accounted for tumor initiation, progression, metastasis, relapse and therapeutic resistance. Ubiquitination and deubiquitination of stemness-related proteins are essential for CSC maintenance and differentiation, even leading to execute various stem cell fate choices. Deubiquitinating enzymes (DUBs), specifically disassembling ubiquitin chains, are important to maintain the balance between ubiquitination and deubiquitination. In this review, we have focused on the DUBs regulation of stem cell fate determination. For example, we discuss deubiquitinase inhibition may lead stem cell transcription factors and CSCs-related protein degradation. Also, CSCs microenvironment is regulated by DUBs activity. Our review provides a new insight into DUBs activity by emphasizing their cellular role in regulating stem cell fate and illustrates the opportunities for the application of DUBs inhibitors in the CSC-targeted therapy.

## Background

**Fig. 1:**
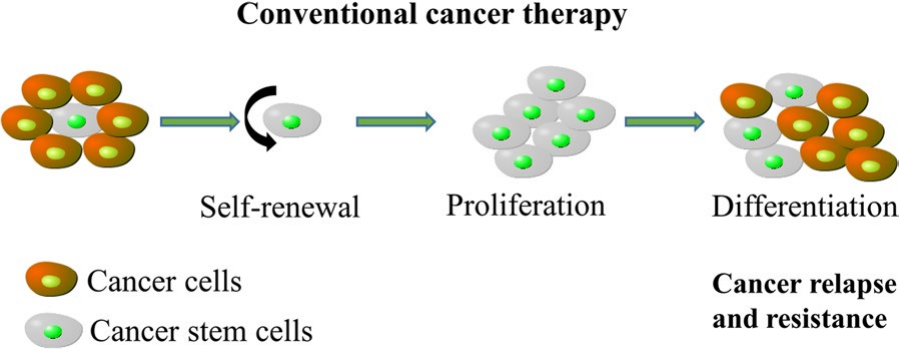
CSCs cause cancer relapse and resistance after conventional cancer therapy. The conventional therapy targeting the tumor bulk without targeting the CSCs leads to tumor recurrence

The existence of cancer stem cells (CSCs) are considered to play a pivotal role in tumor recurrence, resistance and progression [1, 2]. There are three main aspects to effect CSCs maintenance and differentiation, including transcription factor network, CSC-related proteins and microenvironment [3, 4]. Conventional cancer therapy can’t kill cancer stem cells, which will cause cancer relapse and drug resistance under certain conditions (Fig. 1).

**Fig. 2:**
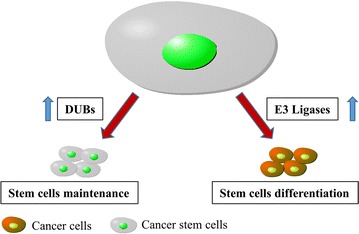
Regulating CSCs differentiation and pluripotency by ubiquitination and deubiquitination. Ubiquitination of core stem cell transcription factors or related key proteins by E3 ligases may drive CSCs differentiation, but deubiquitination of those proteins by DUBs mediates stem pluripotency

Ubiquitination is a post-translational modification process that participates in the covalent conjugation of small, highly conserved 76 amino acid protein ubiquitin with the lysine residues of the substrate protein through the cascade of enzyme reactions, including E1-activating enzymes, E2-conjugating enzymes, and E3 ligases, resulting in protein final degradation, relocalization or activity change. On the contrary, DUB-mediated deubiquitination removes the ubiquitin labels to protect substrate proteins from above-mentioned changes caused by ubiquitination. It has been reported that the ubiquitination and deubiquitination of the key proteins in stem cells may determine the fate of cells (Fig. 2). Recently, DUBs have been demonstrated as promising targets for cancer therapy [5–7], their functions in cancer cell stemness remains elusive. For example, USP54 is overexpressed in colorectal cancer stem cells and promotes intestinal tumorigenesis [8]. USP28 confers stem-cell-like traits to breast cancer cells [9].

Finding deubiquitinates of transcription factors and key protein can provide better understand of the activation mechanism on CSCs, and further deubiquitination inhibitors can be used to eliminate CSCs in cancer radical treatment.

## DUBs and CSC-associated transcription factors

Embryonic stem cells (ESCs) self-renewal and differentiation are known to be regulated by a network of transcription factors including Oct3/4, Sox2, c-Myc, Klf4 and Nanog [10, 11]. Cancer stem cells share significant similarity with normal stem cells in biological characteristics such as quiescence, self-renewal and differentiation [12, 13].

### Sox2

Sox2 also regulates the differentiation and stemness in cancer stem cells [14]. USP22 is located directly on the Sox2 promoter and negatively regulates Sox2 transcription in ESCs [15]. In brain tumor cells, Usp9x was associated with Sox2 and played key roles in the growth of tumor cells, but the relationship between them was not clear [16]. Sox2 also regulated DUBs activity by binding to the promoter region at the transcriptional level, such as USP7, USP25, USP37, and USP44 [17].

### c-Myc

c-Myc is a classical CSC-related marker, which can be stabilized by many DUBs. USP37 directly deubiquitinates and stabilizes c-Myc in lung cancer [18]. USP22 positively regulates c-Myc stability and tumorigenic activity in mammalian and breast cancer cells [19]. In a subset of human breast and lung cancers, USP36 interacts with and deubiquitinates c-Myc [20]. USP28 is required for c-Myc stability in human tumor cells, which binds to c-Myc through an interaction with FBW7alpha, an F-box protein that is part of an SCF-type ubiquitin ligase [21].

### Nanog and ID proteins

Recent studies demonstrated that USP21 maintained the stemness of mouse embryonic stem cells via stabilization of Nanog by removing K48-linked ubiquitin chains [22]. Inhibitor of DNA binding (ID) proteins are transcriptional regulators that control the timing of cell fate determination and differentiation in stem and progenitor cells during normal development and adult life [23]. The small molecule inhibitor of USP1 promotes ID1 degradation and has cytotoxicity to leukemic cells [24]. USP1 deubiquitinated and stabilized ID1, ID2, and ID3 proteins to preserve a mesenchymal stem cell program in osteosarcoma [25].

Some pluripotent factors such as Oct3/4, Klf4 and Lin28 have not been found their DUBs, but all of them are affected by the 26S proteasome, suggesting a potential role of DUB for their stabilization in CSCs.

## DUBs and CSC-related proteins

Some CSC-related proteins also control the fate of CSC, such as SIRT1, P53, PTEN, LSD1, PRC and so on. SIRT1, a NAD^+^-dependent histone deacetylase, influences stem cell aging by controlling mitochondrial biogenesis and turnover which may be required for self-renewal [26, 27].

### SIRT1

SIRT1 inhibition represents a potential approach to target leukemia stem cells [28, 29]. USP22 interacts with and stabilizes SIRT1 by removing polyubiquitin chains conjugated onto SIRT1 in mouse embryonic development [30].

### P53

P53, tumor suppresser, demonstrates a role for p53 deficiency in enhancing the formation of tumors arising from stem cells (embryonal carcinoma cells) [31, 32]. It is reported that USP10 deubiquitinates p53, reversing Mdm2-induced p53 nuclear export and degradation [33]. Ataxin-3, the machado–joseph disease deubiquitinase, interacts with p53 and functions as a novel p53 DUB [34]. USP7 deubiquitinates both p53 and MDM2, one of the ubiquitin ligases that ubiquitylates p53, thereby stabilizing both proteins [35, 36]. OTUD1, OTUD5 and USP11 directly deubiquitinating p53 and functional proteins were required for p53 stabilization [37–39].

### PTEN

PTEN loss leads to the development of cancer stem cells, with the capacity of self-renewal and multi-lineage differentiation [40–43]. ATXN3 acts primarily by repressing PTEN transcription, without altering PTEN protein stability [44]. However, USP18 overexpression could stabilize PTEN protein, and USP18 repression decreases mainly cytoplasmic PTEN [45]. PTEN subcellular compartmentalization can be regulated by USP7 [46, 47].

### PRC

The dysfunction of polycomb repressive complex (PRC) is closely related to cancer stemness [48, 49]. PRC1 represses transcription is only in part dependent on its ubiquitination activity, and Fbxl10 is reported to recruit PRC1 to CpG islands and regulate H2A ubiquitylation [50, 51]. Polycomb gene silencing may require H2A ubiquitination by PRC1 and H2A deubiquitination by Polycomb repressive deubiquitinase (PR-DUB). In some cancer types, PRC1 can be deubiquitinated by USP7, USP11 and USP26 [52, 53]. PRC2-mediated histone methylation plays an important role in aberrant cancer gene silencing and is a potential target for cancer therapy. The PRC2 proteins EZH2 is frequently overexpressed in mesothelioma with BAP1 mutation [54]. The deubiquitination enzymes of PRC need to be further explored in the future.

### LSD

Lysine-specific demethylase 1 (LSD1), the first identified histone demethylase, maintains cell stemness during cancer progression [55, 56]. USP7 and USP28 inhibited LSD1 ubiquitination and stabilized LSD1 protein level [9, 57].

Taken together, CSC-related proteins degradation or activity inhibition by targeting DUBs is effective for eliminating cancer stem cells.

## DUBs and CSC microenvironment

The microenvironment of CSC has also been reported to play essential roles in maintenance of cancer stemness. Tumor specific microenvironments comprise stromal cells, immune cells, networks of cytokines and growth factors, hypoxic regions, and the extracellular matrix (ECM). We summarize the role of CSC microenvironment from two aspects: hypoxia and inflammation [58–60].

### Hypoxia

Hypoxia is considered to be a major feature of the tumor microenvironment and is a potential contributor to the CSC phenotype. Hypoxia-inducible factor (HIF) transcription factors (HIF-1α and HIF-2α) are key mediators in cancer hypoxia response and help maintain multiple CSC population [61, 62]. In the presence of oxygen, VHL tumor suppressor protein interacts with HIF proteins and this interaction results in the ubiquitination and degradation of HIF proteins, maintaining low levels of these transcription factors [63]. However, HIF proteins stabilization can be regulated by DUBs, such as USP8, USP19 and USP28 [64–66]. In addition, USP52 is a key component of P-bodies required to prevent HIF1α mRNA degradation [67].

### Inflammation

The inflammatory cytokines modify the cancer microenvironment, CSCs secretion factors attract the necessary cells into their areas, enabling them better survive and escape chemotherapy [68]. Transforming growth factor β (TGFβ) has the ability to regulate immune cell populations in inhibiting and promoting tumor formation and progression active [69]. Cancer cells exposed to IL-6 are malignant, such as enhanced invasive ability and drug resistance [70, 71]. IL-8 promotes angiogenic activity through the activation of VEGFR2 [78]. USP21 binds to the promoter region of IL-8 and mediates transcriptional initiation in stem-cell like property of human renal cell carcinoma [79]. Also, IL-6 and G-CSF levels have been elevated in lung CSCs [80]. Most inflammatory cytokines are produced by many kinds of signal pathways and the deubiquitination of key proteins in the pathway can block inflammatory cytokines release. For example, TRAF6, a key regulator in toll-like receptor pathway and NF-κB pathway, can be regulated by USP4 and A20 [81, 82].

## Conclusions

CSCs are difficult to eliminate by conventional treatment, mainly due to disorders of signal transduction and epigenetics. The control of ubiquitination and deubiquitination of CSC-related proteins determine the difference in CSCs and the maintenance of pluripotency. DUBs can protect the stemness of the CSC, thereby maintaining its activity and further forming a vicious circle. Therefore, DUBs are very important in the CSC specific treatment. We summarized the effect of deubiquitinating enzymes in the regulation of target proteins in Table 1. The successful inhibition of CSC maintenance and radiation resistance by USP1 specific inhibitor (pimozide) has been provided the basis for further clinical trials [83]. It means that DUB inhibitors may boost more advantages in CSC-specific therapy than other anti-cancer drugs such as proteasome inhibitors. For example, b-AP15, a selective DUB inhibitor, can overcome bortezomib resistance in multiple myeloma [84]. More relevant basic research should be carried out to determine the DUBs related to the CSCs and to identify the mechanisms between them. Currently commercialized DUB inhibitors are summarized in Table 2, showing significant pharmacological effects on cancer cells or cancer stem cells. In general, strategies involving the use of DUB inhibitors to target combination therapy of cancer stem cells and differentiated cancer cells can provide better outcomes for radical cancer treatment.

**Table 1:** The effect of deubiquitinating enzymes in the regulation of target proteins

Proteins	Deubiquitinating enzymes	Effect	References
Sox2	USP22	Transcription	[15]
	USP9X	Unclear	[16]
c-myc	USP37	Protein stabilization	[18]
	USP22		[19]
	USP36		[20]
	USP28		[21]
Nanog	USP21	Protein stabilization	[22]
ID proteins	USP1	Protein stabilization	[24, 25]
SIRT1	USP22	Protein stabilization	[30]
p53	USP10	Protein stabilization	[33]
	Ataxin-3		[34]
	USP7		[35, 36]
	OTUD1		[37]
	OTUD5		[38]
	USP11		[39]
PTEN	ATXN3	Transcription	[44]
	USP18	Protein stabilization	[45]
	USP7	Location	[46, 47]
PRC1	USP7	Protein stabilization	[52]
	USP11		[53]
	USP26		[77]
PRC2	BAP1	Unclear	[54]
LSD1	USP7	Protein stabilization	[57]
	USP28		[9]
HIF-1α	USP8	Protein stabilization	[66]
	USP19		[65]
	USP28		[64]
	USP52	mRNA degradation	[67]
IL-8	USP21	Transcription	[79]
TRAF6	USP4	Activity	[81]
	A20		[82]

**Table 2:** DUB inhibitors for preclinical application in CSC-targeted therapy

Inhibitors	Targeted DUBs	CSC type	References
Pimozide	USP1	Osteosarcoma, glioblastoma	[25, 83]
ML323	USP1		
P5091	USP7, USP47	Neural, glioblastoma, multiple myeloma	[57, 85–87]
P22077	USP7, USP47		
WP1130	USP9x, USP5, UCHL1, USP14, UCH37	Liver, breast cancer	[72, 73]
IU1	USP14	Gastric, multiple myeloma	[74, 75]
b-AP15	USP14, UCHL5		
VLX1570	USP14		
LDN-57444	UCHL1, UCHL3	Prostate	[76]
TCID	UCHL3, UCHL5	Multiple myeloma	[84]

## Abbreviations

CSCs: cancer stem cells
DUBs: deubiquitinating enzymes
ESCs: embryonic stem cells
ID: inhibitor of DNA binding
PRC: polycomb repressive complex
LSD1: lysine-specific demethylase 1
ECM: extracellular matrix
HIF: hypoxia-inducible factor
TGFβ: transforming growth factor β
